# Machine learning-based fracturing parameter optimization for horizontal wells in Panke field shale oil

**DOI:** 10.1038/s41598-024-56660-8

**Published:** 2024-03-13

**Authors:** Weirong Li, Tianyang Zhang, Xinju Liu, Zhenzhen Dong, Guoqing Dong, Shihao Qian, Zhanrong Yang, Lu Zou, Keze Lin, Tao Zhang

**Affiliations:** 1 Xi’an Shiyou University, Xi’an, 710065 China; 2 China University of Petroleum (Beijing), Beijing, 102249 China; 3 Petrochina Changqing Oilfield Company, Xi’an, 710200 China

**Keywords:** Machine learning, Shale oil, Fracturing parameter optimization, Sensitivity analysis, Integrating reservoir simulation, Energy science and technology, Mathematics and computing

## Abstract

In the process of developing tight oil and gas reservoirs, multistage fractured horizontal wells (NFHWs) can greatly increase the production rate, and the optimal design of its fracturing parameters is also an important means to further increase the production rate. Accurate production prediction is essential for the formulation of effective development strategies and development plans before and during project execution. In this study, a novel workflow incorporating machine learning (ML) and particle swarm optimization algorithms (PSO) is proposed to predict the production rate of multi-stage fractured horizontal wells in tight reservoirs and optimize the fracturing parameters. The researchers conducted 10,000 numerical simulation experiments to build a complete training and validation dataset, based on which five machine learning production prediction models were developed. As input variables for yield prediction, eight key factors affecting yield were selected. The results of the study show that among the five models, the random forest (RF) model best establishes the mapping relationship between feature variables and yield. After verifying the validity of the Random Forest-based yield prediction model, the researchers combined it with the particle swarm optimization algorithm to determine the optimal combination of fracturing parameters under the condition of maximizing the net present value. A hybrid model, called ML-PSO, is proposed to overcome the limitations of current production forecasting studies, which are difficult to maximize economic returns and optimize the fracturing scheme based on operator preferences (e.g., target NPV). The designed workflow can not only accurately and efficiently predict the production of multi-stage fractured horizontal wells in real-time, but also be used as a parameter selection tool to optimize the fracture design. This study promotes data-driven decision-making for oil and gas development, and its tight reservoir production forecasts provide the basis for accurate forecasting models for the oil and gas industry.

## Introduction

In recent years, unconventional resources have received increasing attention due to the continuous demand for fossil fuels. The success of multistage fractured horizontal wells (MFHW) has unlocked unconventional oil and gas resources with remarkable results worldwide^1^. Horizontal wells are hydraulically fractured to form a certain geometry of proppant fractures in the reservoir and ultimately achieve increased production. Thus, an accurate forecast of the production performance of MFHW is critical for production optimization, and fracturing parameters optimization is extremely important before the operation of unconventional reservoirs. However, both tasks are still tremendous challenges due to the complicated fluid transport mechanism in fractured unconventional reservoirs and the complex influencing factors.

Various technical solutions have been proposed to predict production from unconventional reservoirs, including decline curve analysis (DCA), material balance equations (MBE), analytical simulations, numerical simulation, and emerging machine learning techniques. DCA and its various modifications, such as the power law exponential (PLE) method^2^, stretched exponential decline (SEDM) method^3^, and Duong’s method^4^, are widely used for forecast production performance of unconventional resources due to their handiness. However, these approaches only require production data and history and cannot account for dynamic variations of good operations and fracturing treatments. MBE methods can be applied to obtain gas content, in-place resources, and estimated ultimate recovery (EUR) in unconventional reservoirs, but their reliability decreases with complicated pressure conditions. Analytical simulation can correlate production data with well-operation conditions, but they fail to take into account of plex unconventional reservoir mechanisms, such as phase transition, by making some simple assumptions. Great efforts have been made to simulate complex mechanisms (including stress sensitivity and non-Darcy flow) as well as fracture models (e.g., the Embedded Discrete Fracture Model (EDFM))^5,6^ to more effectively characterize fracture in unconventional formations. Although numerical simulations can achieve relatively accurate yields, they usually demand a huge amount of computational time due to resolving mass partial differential equations. In the era of rapid advancements in data science, the application of machine learning algorithms has become prevalent in production prediction. These algorithms include methodologies like support vector machine (SVM)^7^, fuzzy logic (FL)^8^, neural networks^9–11^, and the decision tree algorithm^12^.

The optimization of fracturing parameters of MFHWs based on machine learning is an important guideline to improve productivity. Many scholars have done in-depth research and analysis on this subject and have made great progress. Liao et al.^13^ used the BP neural network algorithm to train and learn data from fractured wells with formation parameters, fracturing parameters, and workover parameters as input parameters to derive the relationship curve between sand use and post-fracturing production. In a study conducted by Zhou et al.^14^, traditional regression techniques were employed to investigate the connection between well dynamics and completion attributes. The objective was to ascertain the production behavior of Marcellus shale oil and gas wells. The study's findings indicated a robust correlation between well dynamics and factors such as the number of hydraulic fracture stages and lateral length.

Lolon et al.^15^ formulated diverse models, including multiple regression, random forest, and gradient boosting, to analyze the connection between well parameters and the overall oil production in horizontal wells located within the Middle Bakken and Three Forks formations. The outcomes of their research indicated that water content emerged as the foremost predictor of cumulative oil production. Additionally, among the completion parameters, total fracturing fluid and pumped proppant were identified as the most significant factors in predicting oil production. In their study, Luo et al.^16^ performed an extensive analysis of a dataset encompassing approximately 2,000 fractured horizontal wells within the Bakken shale oil region. The researchers employed three distinct approaches, namely random forest, recursive feature elimination, and Lasso regularization, to assess the key factors influencing yearly oil production. Additionally, an artificial neural network was employed to develop a predictive model for annual oil production. The intention behind these analyses was to enhance well operations through optimization. In their research, Clar et al.^17^ harnessed an artificial neural network to anticipate the production outcomes of horizontal wells within the shale oil reserves of the Eagle Ford region. Their study revealed substantial correlations between total production and several variables, including lateral length, vertical depth, porosity, and the volume of fracturing fluid. Duplyakov et al.^18^ used a data-driven model for the optimal design of hydraulic fracturing parameters, a hydraulic fracturing database was established using data from 22 fields in Siberia, Russia, and a predictive production model was developed for fracturing optimization design. Yuwei et al.^19,20^ proposed a rock brittleness evaluation method based on the statistical constitutive relationship of rock damage, which laid the analytical foundation for hydraulic fracturing. Subsequently, a mathematical model of hydraulic fracture height for high-stress and multi-layered complex formations was developed and solved for predicting the fracture height in hydraulic fracturing. Temoor et al.^21^ using three new socially-inspired algorithms, combined with reservoir simulation and artificial neural networks, the hydraulic fracturing design parameters were successfully optimized to improve the tight gas production performance, which performed better compared to traditional optimizers. Dong et al.^22^ by combining machine learning with evolutionary algorithms, based on a large number of static and dynamic datasets, the production prediction model is established using machine learning methods, and the fracturing parameters are optimized by particle swarm optimization algorithms, which provide effective fracturing design for tight reservoir production.

At the same time, machine learning technology has its limitations in application. For example, in some application scenarios, especially decisions involving important factors such as security and the environment, the interpretability of the model is critical. However, the complexity and black-box nature of machine learning models make it difficult for non-machine learning professionals to understand their decision-making processes, which may limit their application in practical engineering. In addition, machine learning lies in the process of combining numerical simulation. The accuracy of parameters obtained by numerical simulation model, the challenge of integrating and coupling machine learning model with numerical simulation model and the reliability of machine learning model are all potential problems. The time cost and resource cost of calculation are also aspects that need to be considered in practical applications.

Although progress has been made in optimizing hydraulic fracturing parameters in horizontal wells, many difficulties and challenges have been encountered^23^. Given the multitude of factors that influence the effectiveness of fracturing, such as geological characteristics, fracturing conditions, production dynamics, and other variables^24^, the interplay between these parameters is intricate and intricate. It's important to note that the connection between diverse parameters and the impact of fracturing is not merely linear in nature. Conventional numerical simulation approaches exhibit certain limitations, including extended computational durations, imprecise representation of fracture networks, and a singular seepage mechanism^25^. These limitations result in the generation of extensive and intricate datasets throughout the phases of fracturing construction and production. The study of fracturing parameters optimization of MFHWs based on machine learning techniques requires a large amount of existing well data as training data, which has the problems of big data volume and high cost. The current research focuses mainly on the analysis of master control factors of production, production prediction, and prediction of other related parameters, while there are few studies account for the economic benefits of fracturing schemes.

In this study, we propose a complete workflow for optimizing the fracture parameters of MFHWs, combining reservoir numerical simulation with machine learning techniques to generate a proxy model, using PSO to optimize fracture parameters with maximizing NPV, ultimately improving the economic benefits of unconventional reservoirs. Compared to traditional reservoir simulation methods, the time required to train a good proxy model by machine learning method is extremely short. Furthermore, it renders practical subsequent optimization of horizontal well fracturing parameters efficiently and quickly under a variety of conditions.

The structure of the rest of this document is as follows: In Section "Methodology", we present the approach for assembling a training dataset, predicting shale oil production, and fine-tuning fracturing parameters. Moving on to Section "Results", we implement this workflow on two specific cases and outline the key findings. In Section "Discussions and future work", we delve into the limitations and prospects for future research, and lastly, Section "Conclusions" provides the concluding remarks for this study.

## Methodology

**Figure 1:**
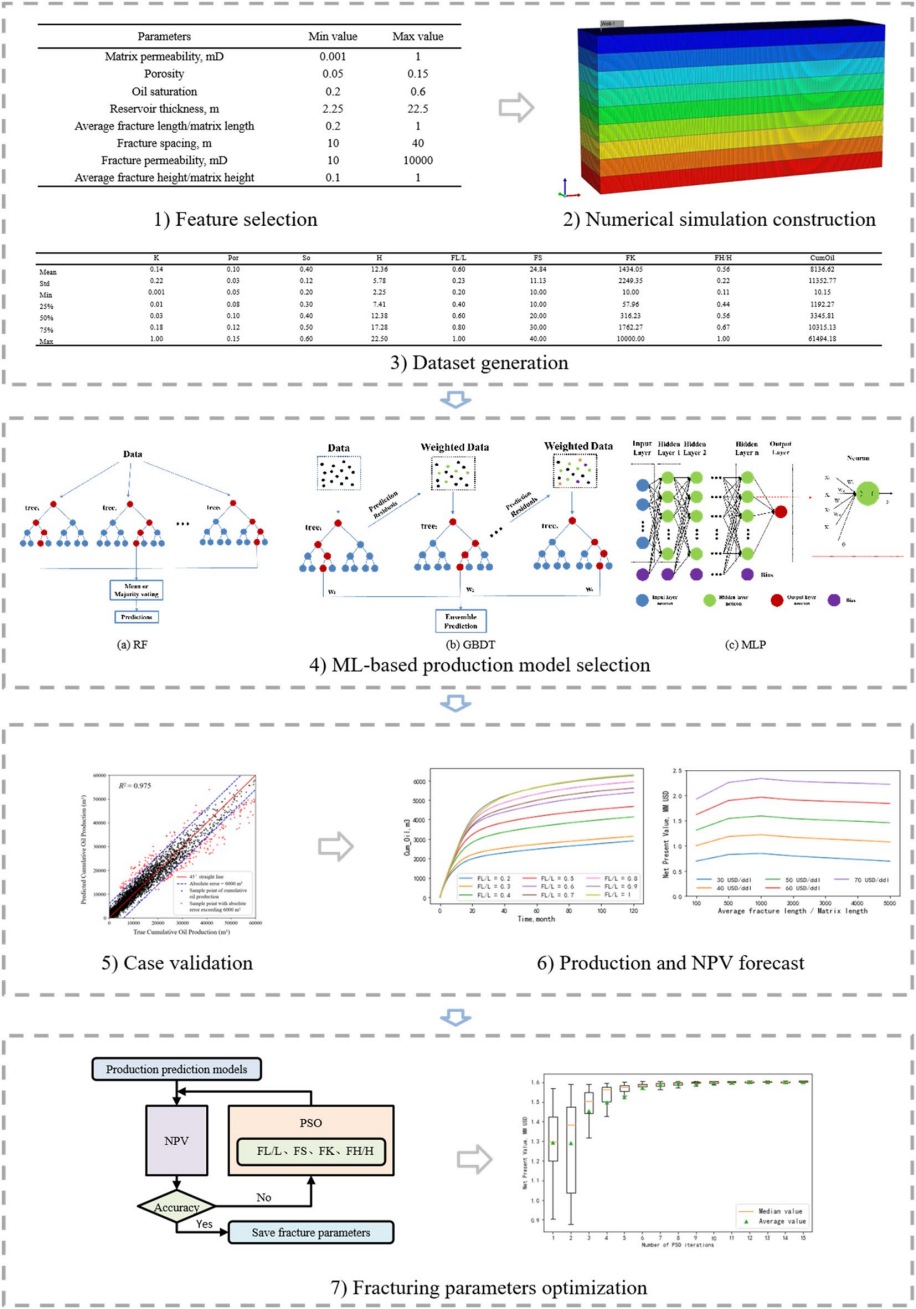
Workflow for optimization fracturing parameters of tight oil reservoirs using machine learning.

Figure 1 shows our workflow to predict tight oil production and optimize the fracturing design. It consists of seven steps: (1) *Data preparation*. Select the characteristic parameters that have a more obvious impact on productivity; (2) *Numerical simulation construction*. Construct a representative numerical model of MFHW in a tight oil reservoir; (3) *Data generation*. Perform numerical simulations to construct the samples for training production forecast model; (4) *Production forecast model select*. Develop multiple machine learning (ML) models, undergo training, and subsequently assess the efficacy and resilience of these ML-based models; (5) *Case vaildtion*. Identify the most optimal ML model as a proxy tool for forecasting tight oil production; (6) *Forecast production*. Validate the selected ML model through the real case, forecast tight oil production and NPV; 7) *Fracturing parameter optimization*. Optimize the selected fracturing parameters with PSO algorithm.

### Data preparation and generation

Constructing a data-driven model necessitates a significant array of geological attributes and well-completion data as inputs. Subsequently, machine learning techniques are applied to establish connections between reservoir characteristics and production trends. Ideally, the acquisition of datasets from actual fields is preferable for constructing these data-driven models. However, data collected from oilfields usually cause some issues, including data missing, format errors, and intricate sources, and might lead to complications in their direct usage. Demonstrably, synthetic data produced through numerical or analytical models can serve as an alternative when there is a scarcity or absence of high-quality real-world data^26^.

**Figure 2:**
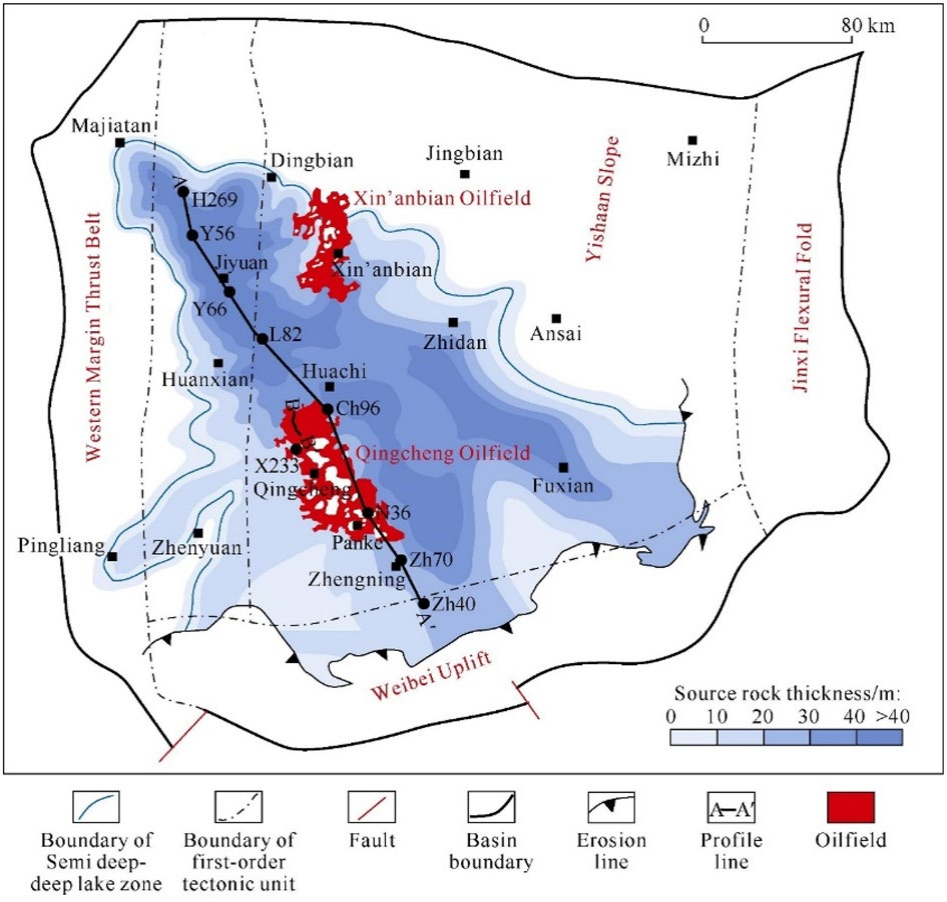
Lake basin distribution and tectonic division during depositional period of Chang 7 Member^28^.

Reservoir and fluid parameters from the Chang 7 tight oil reservoir within the Triassic Extension Formation at the Panke area of the Ordos Basin are gathered for the construction of numerical models. Notably, this reservoir holds a prominent position as one of China's major tight oil fields. Positioned at the east–west tectonic junction in China, the Ordos Basin was a component of the North China Basin during the Paleozoic era. Within this basin, the Triassic Extension Formation is characterized by a series of clastic rock systems from inland river deltas and lacustrine environments. This formation is further categorized into 10 distinct sections, proceeding from the uppermost to the lowermost layers^27^. As illustrated in Fig. 2, the Chang 7 formation is primarily composed of mudstone, with a sand content of less than 20%. This formation is further subdivided into three distinct subsections (Chang 7–3, Chang 7–2, Chang 7–1) progressing from the bottom to the top layers. In general, the Chang 7 formation exhibits suboptimal reservoir properties. Porosity levels span between 2.07% and 18.75%, averaging at 10.77%, while permeability ranges from 0.03 mD to 3.23 mD, with an average of 0.38 mD. This reservoir typically displays low porosity and remarkably low permeability, although it commonly manifests well-developed microfractures.

**Figure 3:**
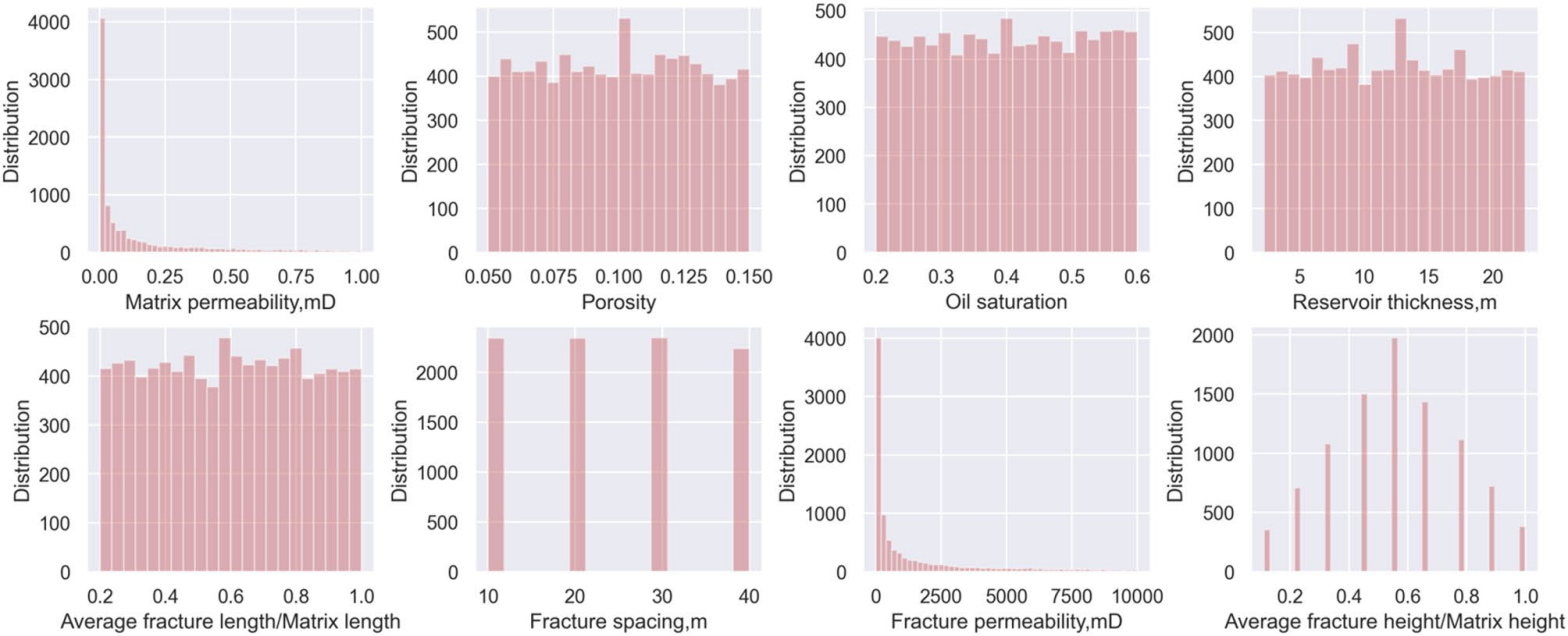
Histograms of key input features to the model.

The dataset for training the ML-based production model accounts for various reservoir features and their corresponding tight oil production. Initially, we opt for pivotal reservoir attributes that influence tight oil production and employ them as features for generating the dataset. Based on previous studies^29,30^, we select and combine eight factors that affect tight oil production the most as features, including porosity, oil saturation, reservoir thickness, matrix permeability, fracture permeability, fracture spacing, average fracture length/matrix length, and average fracture height/matrix height. The first three features are the main characteristics that determine the oil and gas in-place resource. Matrix and fracture permeability are dominant factors affecting production performance and should be set separately. The other selected features that account for fracturing treatment as comprehensively as possible include fracture spacing, average fracture length/matrix length, and average fracture height/matrix height, as the ML-based model is expected to serve as a powerful tool for production forecast and fracturing design. In Table 1, we summarize the range and distribution type of the eight selected features, which represent the various geological and stimulation conditions of Chang 7 tight oil reservoirs. A total of 10,000 sets of combinations are generated for the eight parameters with the Latin hypercube sampling method (LHS), which is used to generate the approximate distribution from random sampling. The statistics of the experimental dataset are summarized in Table 2, and the histograms of the eight key input parameters used in the numerical model are shown in Fig. 3.

**Table 1:** Parameters and related distributions to construct the input database.

Parameters	Min value	Max value	Distribution type	Symbol
Matrix permeability, mD	0.001	1	lognormal	K
Porosity	0.05	0.15	uniform	Por
Oil saturation	0.2	0.6	uniform	So
Reservoir thickness, m	2.25	22.5	uniform	H
Average fracture length/matrix length	0.2	1	uniform	FL/L
Fracture spacing, m	10	40	uniform	FS
Fracture permeability, mD	10	10,000	lognormal	FK
Average fracture height/matrix height	0.1	1	normal	FH/H

**Table 2:** Statistics of main features.

	K	Por	So	H	FL/L	FS	FK	FH/H
Mean	0.14	0.10	0.40	12.36	0.60	24.84	1434.05	0.56
Std	0.22	0.03	0.12	5.78	0.23	11.13	2249.35	0.22
Min	0.001	0.05	0.20	2.25	0.20	10.00	10.00	0.11
25%	0.01	0.08	0.30	7.41	0.40	10.00	57.96	0.44
50%	0.03	0.10	0.40	12.38	0.60	20.00	316.23	0.56
75%	0.18	0.12	0.50	17.28	0.80	30.00	1762.27	0.67
Max	1.00	0.15	0.60	22.50	1.00	40.00	10,000.00	1.00

K-matrix permeability, mD; Por-porosity, *f*; So-oil saturation,* f*; H-reservoir thickness, m; HL/L-average fracture length/matrix length, *f*; FS-fracture spacing, m; FK-fracture permeability, mD; FH/H-average fracture height/matrix height,* f*.

**Figure 4:**
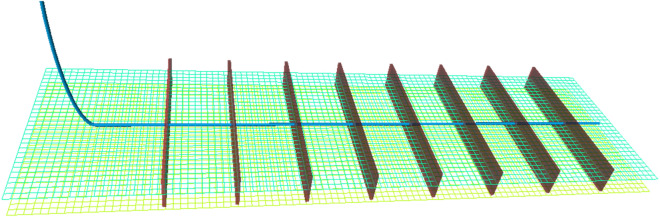
Numerical model to generate the oil and gas production.

**Figure 5:**
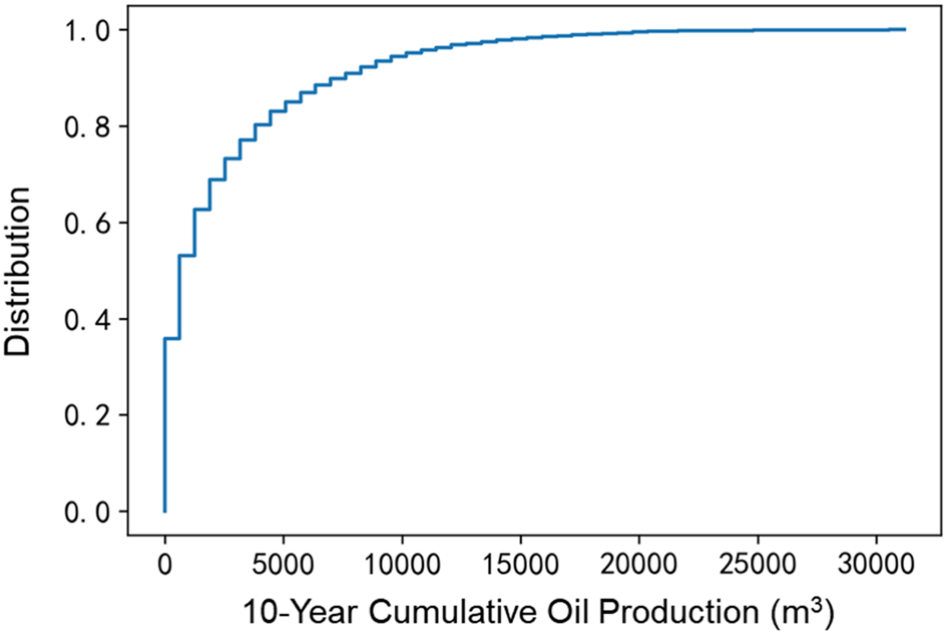
Distribution of the cumulative ten-year oil production.

Then, we apply a commercial numerical simulator (CMG) to construct a multistage fractured horizontal well (MFHW) model and forecast daily tight oil production. The MFHW simulation model is a three-phase, 3D rectangular model. Given the underdeveloped nature of natural fractures in the examined region, a lattice grid is employed to replicate the MFHW (Multi-Fractured Horizontal Well), which is discretized into a grid comprising 200 × 25 × 9 blocks. The horizontal well is positioned at the reservoir's central location and operates by maintaining a consistent bottomhole pressure (BHP). Multistage fractures are set to be perpendicular to the horizontal wellbore. Figure 4 shows a 3D view of the MFHW model. Once the reservoir model is established, the 10,000 sets of input are then populated into it to generate 10,000 simulation cases with CMG simulator. The production span spans a decade, constituting a practical and realistic depiction of tight oil production. As a result, the daily production predictions are obtained to calibrate the ML-based model. Figure 5 shows the cumulative probabilistic distribution of the cumulative ten-year production data.

### Machine learning-based production forecast models

To assess the effectiveness of the ML-based production forecasting model, we first normalized the data. The main purpose of this is to ensure that the range of values for different features is consistent to improve the training and performance of the model. Whereas in this study, we use max–min normalization which scales the data to a specified range, usually [0, 1]. The formula for max–min normalization is as follows:

1 $${{\text{X}}}_{normalized}=\frac{{\text{X}}-{{\text{X}}}_{min}}{{{\text{X}}}_{max}-{{\text{X}}}_{min}}$$

Secondly, we utilize traditional machine learning algorithms for comparison. These include linear regression (LR), support vector regression (SVR), gradient-boosted decision tree (GBDT), random forest (RF), and multilayer perceptron (MLP). LR is one of the most common tools applied in ML for making predictions^31^. LR uses regression analysis in mathematical statistics to demonstrate the quantitative relationship between two or more variables that are dependent on each other. SVR is a particular schema of SVM based on a kernel function for regression^32^. GBDT is characterized by its iterative error reduction during the training phase, making it particularly suitable for addressing imbalances found in real production data^33^. Meanwhile, random forest (RF) acts as a classifier composed of multiple decision trees, and its classification result is determined by the majority outcome among individual trees' outputs^34^. On the other hand, multilayer perceptron (MLP) emulates the information processing capabilities of human brain neurons. It functions as an abstract mathematical model with a distributed parallel information processing approach, exhibiting adaptability and dynamic behavior through a multitude of interconnected simple neurons.

In the experiments, the coefficient of determination (R2) is employed to assess the effectiveness of the ML-based regression model. R^2^ indicates the prediction bias of the proposed model, and a higher value of R^2^ indicates better model performance with a maximum value of 1. The R^2^ value is calculated using the following equation.

2 $${R}^{2}=1-\frac{{{\text{SS}}}_{res}}{{{\text{SS}}}_{tot}}$$

Here, ${{\text{SS}}}_{res}$ denotes the sum of squared residuals, and ${{\text{SS}}}_{tot}$ is the overall sum of squared values, both calculated using the subsequent formula.

3 $${{\text{SS}}}_{res}=\sum {\left({y}_{i}-{y}_{reg}\right)}^{2}$$

4 $${{\text{SS}}}_{tot}=\sum {\left({y}_{i}-\overline{y }\right)}^{2}$$

Here, ${y}_{i}$ represents the value of each data point, $\overline{y }$ signifies the mean value, and ${y}_{reg}$ corresponds to the value projected by the regression model.

The trained ML-based model is applied to forecast the production of MFHW in tight oil reservoirs. The data is dumped into the trained ML-based model, which generates time-serious outputs that represent the production performance. To ensure fair comparisons, identical datasets are utilized for both training and validating the performance of these five models.

### Fracturing parameters optimization

#### Objective function

To validate the superiority of the proposed workflow, we apply the trained DL model to optimize the fracturing parameters. We utilize the net present value (NPV) over the 10-year production span to evaluate the objective function. The NPV calculation is determined by the subsequent formula.

5 $$NPV={\sum }_{t=1}^{n}\begin{array}{c}\frac{{ Q}_{t}*\left({P}_{0}-{C}_{operation}\right) }{{\left(1+r\right)}^{t}}-{C}_{fracturing}-{C}_{oil-testing}-{C}_{other}\\ \end{array}$$

6 $${C}_{t}={Q}_{t}*\left({P}_{0}-{C}_{operation}\right)$$

7 $${C}_{0}={C}_{fracturing}+{C}_{oil-testing}+{C}_{other}$$

where ${C}_{t}$ is the net cash flow in year t, ${Q}_{t}$ is the annual oil production in year t, ${P}_{0}$ is the square oil price, ${C}_{operation}$ is the square oil management cost, ${C}_{0}$ is the initial investment amount, ${C}_{fracturing}$ is the fracturing cost, ${C}_{oil-testing}$ is the test oil cost, ${C}_{other}$ is other costs, $r$ is the base rate of return or discount rate, $n$ is the life cycle of the investment project.

Thus, the mathematical model of objective function used to optimize the fracturing parameters is expressed as

8 $$ max \, NPV\left( {\mathbf{x}} \right) $$

subject to

9 $$l\le \mathbf{x}\le u$$

where **x** refers to a n-dimensional vector consisting of all the variables (*n* equal to four in this study);* l* and *u* are the lower and upper limits of optimization variables, respectively.

The cost and oil price used in this study are shown in Table 3.

**Table 3:** Parameters used for NPV calculation.

Parameters	Details	Unit
Fracturing cost	Base cost: number of fracture × 3 + 15	10^4^ $
	Fluid cost: Fluid volume (m^3^) × 0.005	
	Proppant Cost: Proppant volume (m^3^) × 0.02	
Oil testing cost	Base cost: number of fracture × 1.5 + 2.5	10^4^ $
Other cost	Drilling + Cementing + Logging Cost: Well length × 0.02	10^4^ $
Oil price	60	$/bbl

#### Particle swarm optimization

The optimization of the fracturing parameters is accomplished by employing the PSO algorithm to solve Eqs. (9) and (10). PSO, initially introduced by Eberhart and Kennedy^35^, is an evolutionary computational technique used for this purpose. Its basic concept originated from the study of flock foraging behavior and is a simplified model of a flock intelligence algorithm. The algorithm's initial inspiration stemmed from the patterns observed in the collective behavior of bird flocks in search of prey. This concept led to the development of a simplified model that harnesses swarm intelligence, allowing individuals within the flock to collaborate and share information to collectively determine the optimal solution^36^.

The PSO algorithm's sub-workflow, depicted in Fig. 1, illustrates how each particle conducts an individual search within the defined solution space. The most optimal solution is recorded as the current individual extremum and shared across the entire particle population. As particles traverse the solution space, their speed and position are adaptively adjusted based on their own flight experience and the collective experiences of other particles, thus contributing to their dynamic movement^37^.

The equation used for updating particle velocity in the PSO algorithm is given by:

10 $${{\text{V}}}_{new}=\omega {{\text{V}}}_{id}+{{\text{C}}}_{1}random(\mathrm{0,1})({{\text{P}}}_{id}-{{\text{X}}}_{id})+{{\text{C}}}_{2}random(\mathrm{0,1})({{\text{P}}}_{gd}-{{\text{X}}}_{id})$$

In this context, the elements of the equation are defined as follows: ${{\text{V}}}_{id}$ stands for the current velocity of the particle;$\omega$ represents the inertia factor, signifying the motion inertia associated with velocity; random(0,1) is a function generating random numbers within the range of 0 to 1; ${{\text{P}}}_{id}$ corresponds to the particle's current position; ${{\text{X}}}_{id}$ signifies the global best position of the given particle;${{\text{P}}}_{gd}$ denotes the current best position among all particles within the population; and *C*_1_ and *C*_2_ represent the learning factors, which gather insights from the particle's historical best position and the overall best position across the population.

## Results

### Training of ML models

To verify the effectiveness of the ML-based production prediction models proposed in this study, we apply a *k*-fold technique (*k* = 8) to relieve possible overfitting issues. The k-fold technique enables a comprehensive assessment of ML models by iteratively altering the training and test dataset ratio k times. During each iteration, the training set is employed to build the yield prediction model, while the test set is utilized to validate the model's predictive accuracy and its capacity to generalize to new data. In this study, the hyperparameters for each of the five ML-based models are given in (Table 4).

**Table 4:** Hyper parameters of machine learning models.

ML Model	Hyperparameters
LR	Default
SVR	Default (kernel = "rbf", C = 100, gamma = 0.1, epsilon = 0.1)
GBDT	Default (random_state = 0)
RF	Default (max_depth = 2, random_state = 0)
MLP	Default (random_state = 1, max_iter = 500)

**Figure 6:**
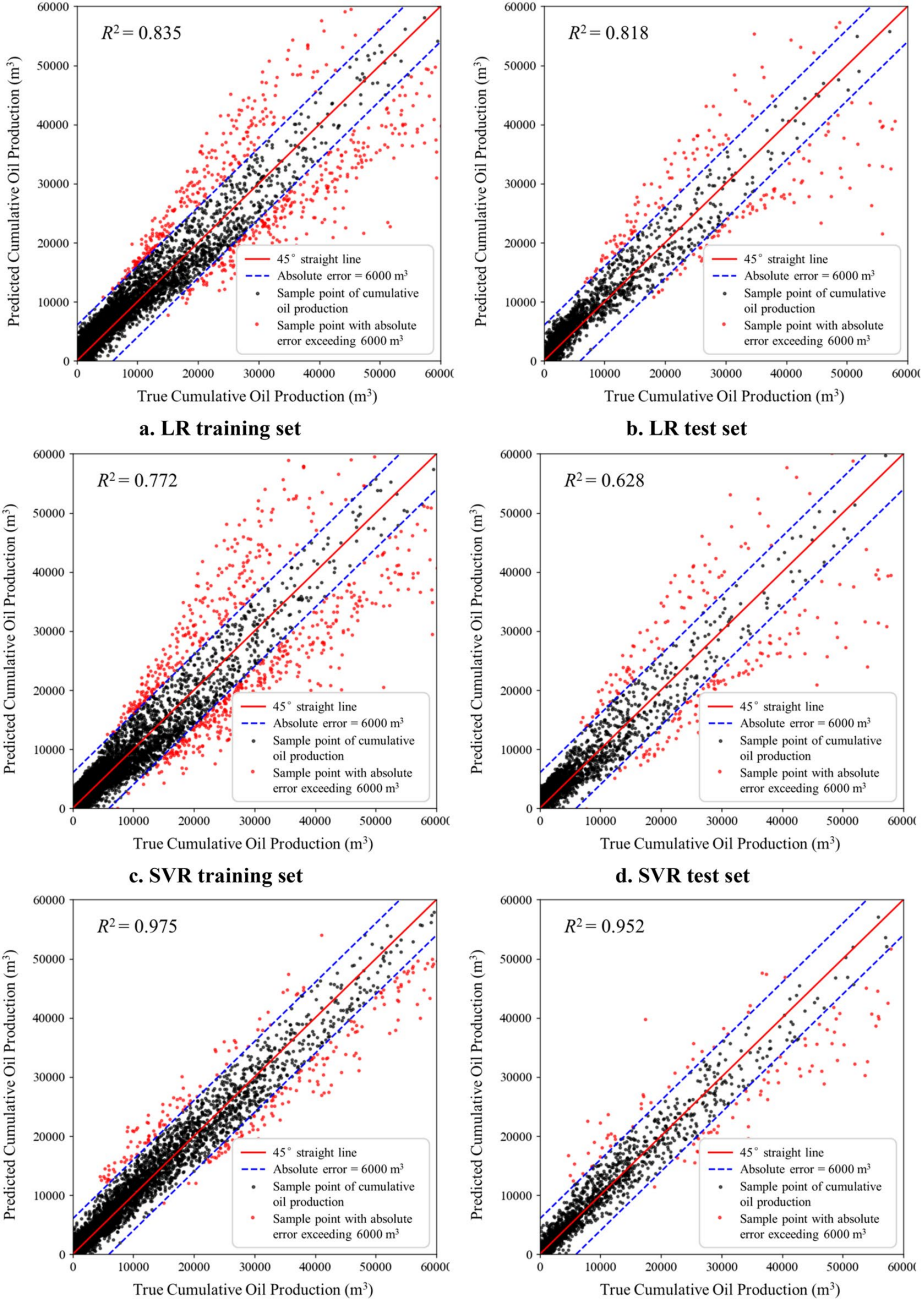

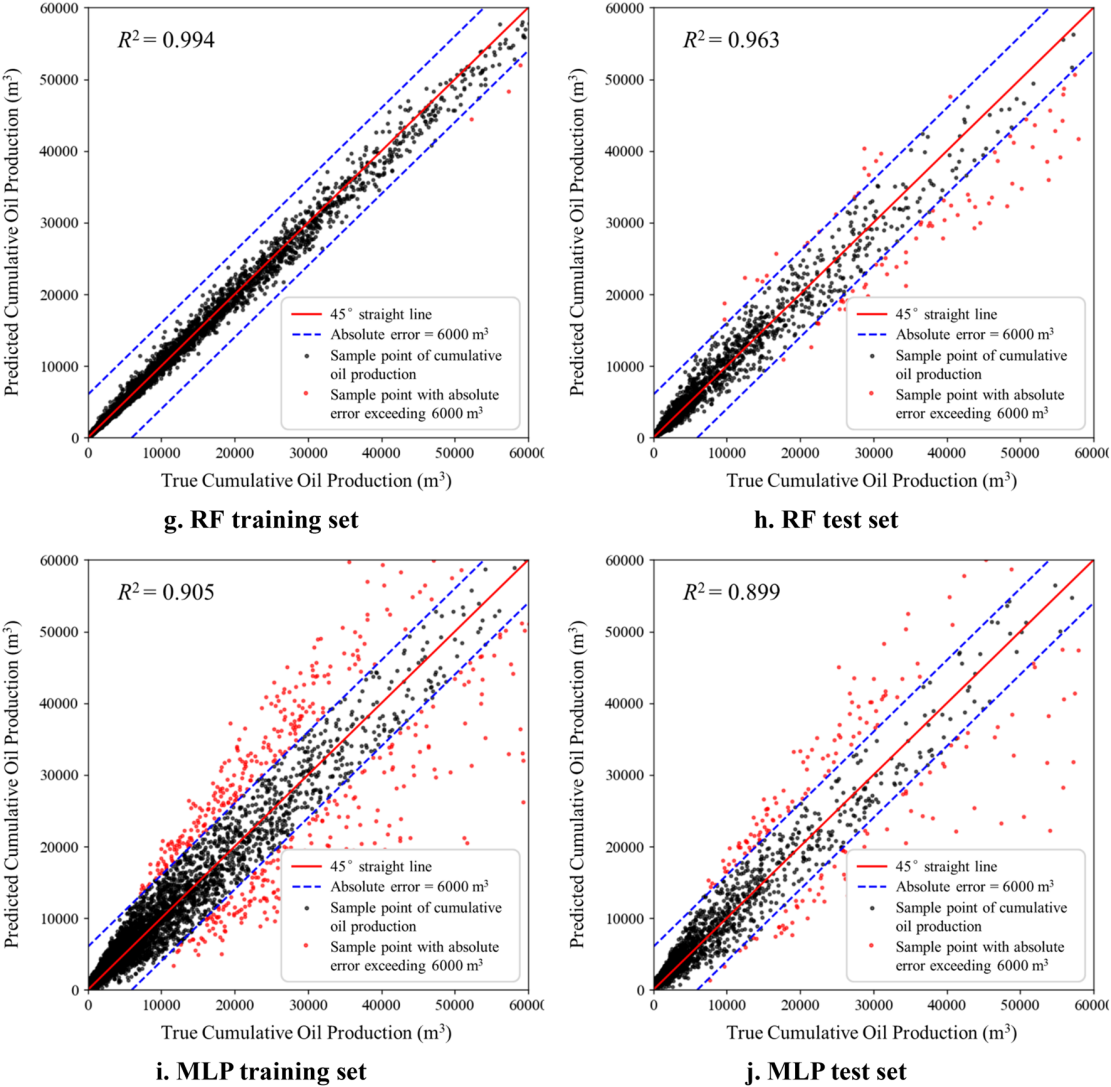
Performance of five machine learning models.

The performance of the five production prediction models is compared using the coefficient of determination (R^2^) as an evaluation index, and then the best machine learning algorithm that applies to the study area can finally be determined. Figure 6 shows the actual cumulative oil production obtained from different numerical simulation cases in the abscissa, and the values predicted by the ML-based production model in the vertical ordinate. The closer the data points lie to the 45° curve, the smaller the errors between the predicted and real samples. The result shows that the random forest model has the best performance among the five machine learning models. Table 5 summarizes the R^2^ of cumulative oil production prediction, taking the mean of the k-fold cross-validation, to compare the prediction accuracy of five production prediction models on the training and test data sets. The metrics still demonstrate the superiority of the RF model, with R^2^ reaching 0.994 and 0.963 for the training and test data sets, respectively. Thus, the random forest prediction model is selected for production prediction and fracturing parameters optimization.

**Table 5:** Evaluation metrics of various ML models.

ML model	R^2^ of training result	R^2^ of test result
LR	0.863	0.818
SVR	0.772	0.628
GBDT	0.975	0.952
RF	0.994	0.963
MLP	0.905	0.899

And also, Table 6 shows the results of MAE and MSE of each model. MAE and MSE tell us the average error degree and error distribution of the model prediction. Together with R^2^ results, the prediction performance of each model is more comprehensively displayed. It can be seen from the results that RF model has become the most reliable model for prediction, scoring 0.006 and 0.012 points respectively in the training set and test set of MSE, and 0.043 and 0.066 points respectively in the training set and test set of MAE, which once again proves the superiority of RF model in our research.

**Table 6:** MSE and MAE metrics of various ML models.

Evaluation	Data set	LR	SVR	GBDT	RF	MLP
MSE	Train set	0.040	0.045	0.011	0.006	0.023
	Test set	0.075	0.091	0.018	0.012	0.064
MAE	Train set	0.093	0.100	0.060	0.043	0.081
	Test set	0.165	0.182	0.082	0.066	0.103

### Real case for Panke tight oil reservoir

**Figure 7:**
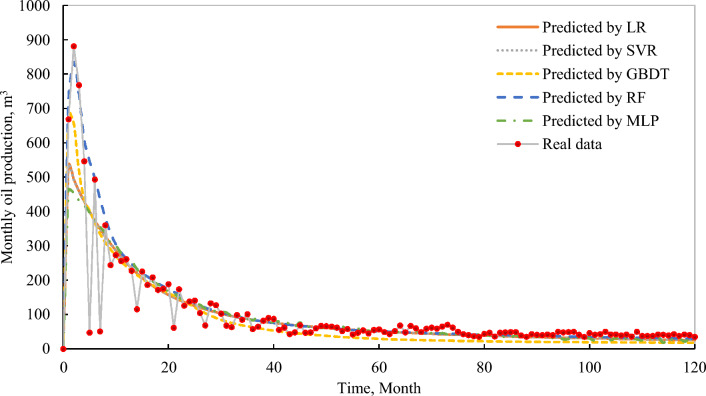
Comparisons between the real production of an MFHW in the Panke tight oil and the prediction using the five machine learning techniques.

Once we train and select the RF model to forecast the tight oil production of MFHWs. We further validate the superiority of the proposed RF model with the actual production historical data of an MFHW in the Panke tight oilfield. The parameters used to predict the production of MFHW and compare it with the actual daily oil production are listed in Table 7. RF model also demonstrates admissible prediction precision and outperforms the other four models (Fig. 7), especially in the phase of early production. Thus, the RF model is a robust alternative to the numerical simulation to speed up the process of optimizing an actual tight oil reservoir.

**Table 7:** Inputs of a real MHFW in the Panke tight oilfield.

Parameter	Unit	Value	Parameter	Unit	Value
Matrix permeability	mD	0.018	Average fracture length	m	350
Matrix porosity	%	0.11	Fracture spacing	m	25
Oil saturation	%	0.42	Fracture permeability	mD	900
Reservoir thickness	m	18.5	Average fracture height	m	15.5

### Sensitivity analysis

The hydraulic fracturing procedure entails injecting a substantial volume of fluid at elevated pressures, inducing rock fractures. Subsequently, a significant quantity of proppant is introduced to sustain open fractures, ensuring their durability and facilitating prolonged high-conductivity pathways^38^. To illustrate a production and NPV improvement tendency, a sensitivity analysis is conducted to demonstrate the effect of various features on output variables (production and NPV). Furthermore, the sensitivity analysis can serve to determine the appropriate range of the fracture parameters for PSO. The sensitivity of different fracture parameters to production is analyzed using the established RF-based production prediction model for MFHWs. The most important fracture stimulation parameters include fracture spacing, fracture permeability, fracture geometry, et al. The initial values and ranges of the different parameters are given according to the actual reservoir conditions (Table 8).

**Table 8:** The range of values for each parameter of the RF-based production prediction model.

Parameters	Initial value	Initial range	Range for PSO
Matrix permeability, mD	0.02	0.02	0.02
Porosity	0.1	0.1	0.1
Oil saturation	0.4	0.4	0.4
Reservoir thickness, m	18	18	18
Average fracture length/matrix length	0.8	[0.2,1]	[0.6,1]
Fracture spacing, m	30	[10,40]	[10, 30]
Fracture permeability, mD	1000	[100,5000]	[500,2000]
Average fracture height/Matrix height	0.8	[0.1,1]	[0.6,1]

#### Cumulative oil production

**Figure 8:**
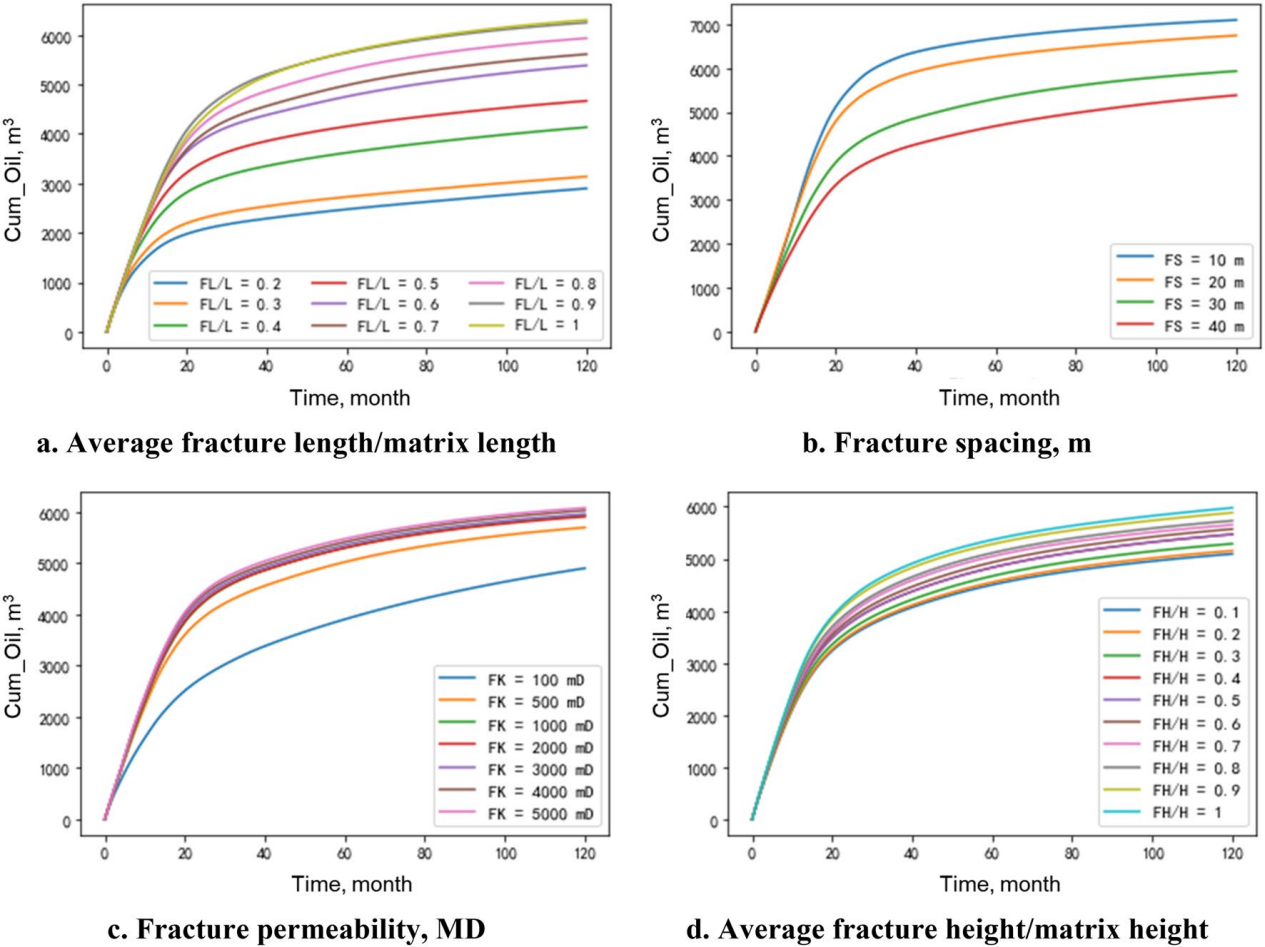
Well production at different fracture parameters.

Figure 8 shows the results of sensitivity analysis, which indicates predicted cumulative production for different values of various fracture parameters as inputs of the RF model. By increasing FL/L (average fracture length/matrix length), the cumulative oil production increases, which shows the model sensitivity to FL/L (Fig. 8a). In Fig. 8b, the influence of FS (Fracture Spacing) on production is illustrated. It's evident that as the FS decreases, oil production experiences an increase. In Fig. 8c, it's apparent that greater fracture permeability leads to a significant rise in well production. However, this increase in oil production tapers off when the permeability surpasses 2,000 mD. This observation serves to narrow down the range of fracture permeability that is subject to further optimization via the PSO technique. Figure 8d indicates that FH/H (average fracture height/matrix height) has less effect on production, and the larger the FH/H, the higher the oil production.

#### NPV

**Figure 9:**
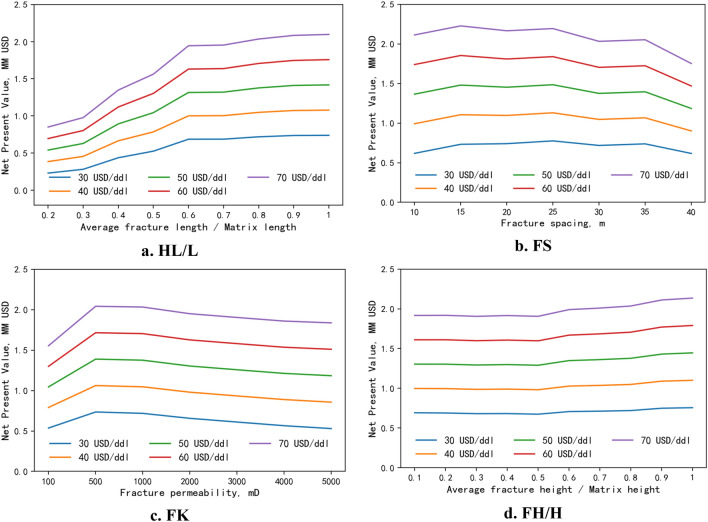
NPV sensitivity of fracture parameters at different oil prices.

For selecting the most appropriate economics index, an operator’s preference is important. Most operators prefer maximum NPV. The sensitivity of different fracture parameters to NPV is analyzed using the RF-based production prediction model. Figure 9a shows the NPV increasing the value of FL/L at different oil prices. When FL/L is greater than 0.6, the effect of FL/L on production gets slighter, which indicates a narrow range for optimizing FL/L. Figure 9b is the effect of FS on NPV, which shows the lower value of FS has a higher NPV. The effect of FK on NPV can be observed in Fig. 9c, which confirms that it has a higher NPV at various oil prices when the permeability is between 500 and 2000. Figure 9d shows the effect of FH/H on NPV, and the increment in NPV is greater when FH/H is larger than 0.8. The narrower ranges of different fracture parameters for further PSO optimization after sensitivity analysis are given in Table 6.

### Case study of fracturing parameters optimization

The core objective of this research is to optimize fracturing parameters ahead of well operations, rather than focusing on predicting the production performance of an MFHW. To achieve this, we employ the extensively used PSO technique. This optimization process relies on the response surface generated through the RF algorithm, applied to a numerical simulation model. The optimized fracturing parameters are based on the sensitivity analysis in Section "Cumulative oil production" to select the parameters that have a greater impact on productivity. The value of other parameters that are not optimization variables remains unchanged. The configurations utilized include 50 particles, a maximum iteration count of 30, and a maximum velocity capped at 3. The learning factors C1 and C2 are set at a value of 2.

**Figure 10:**
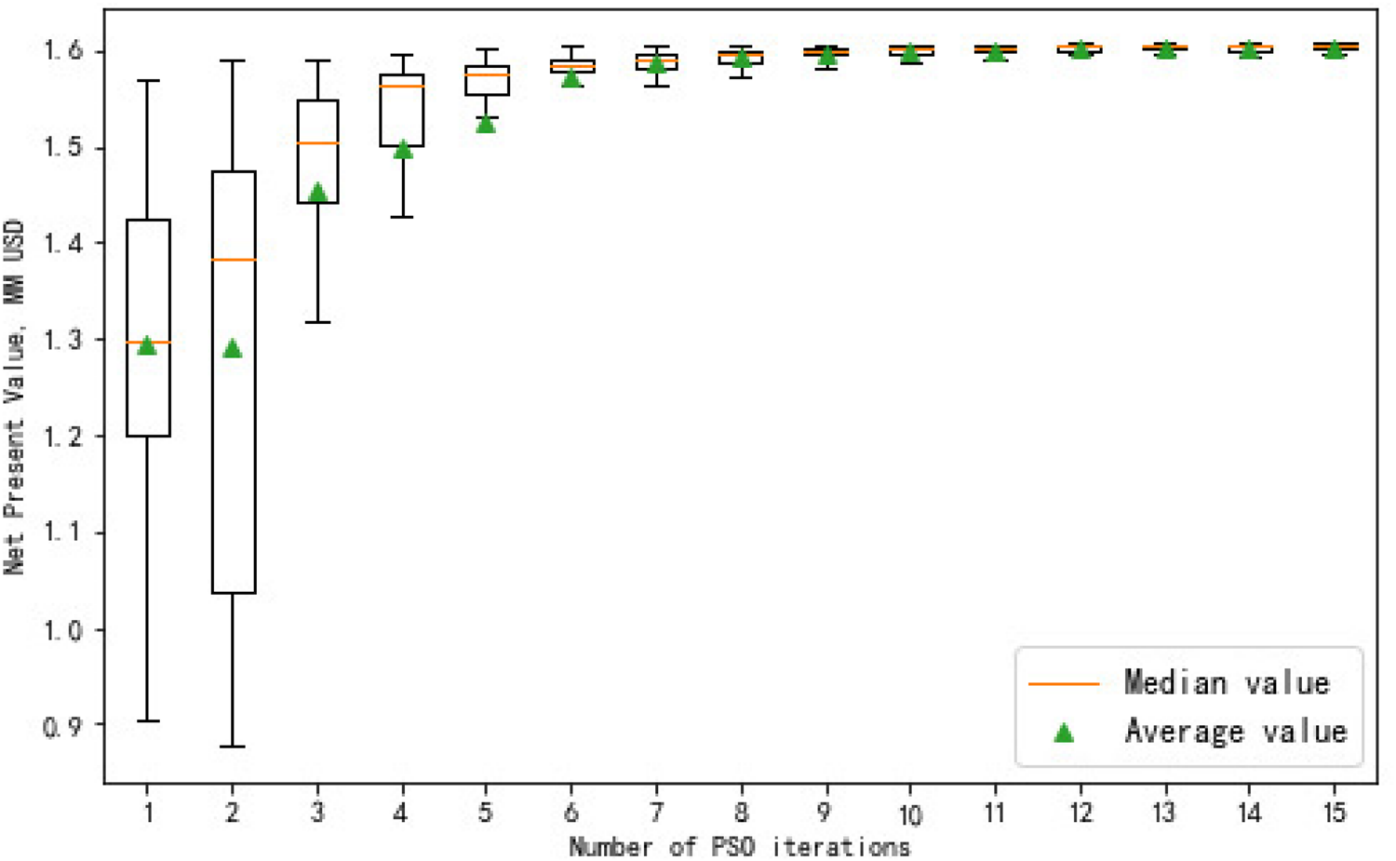
PSO iterations of RF model.

Considering the RF model serves as an alternative to the numerical simulation model, we also calculate the NPV with numerical simulation, whose input features are kept the same as the RF production prediction model. A large number of experiments were conducted to achieve the optimal NPV. The convergence process of the objective functions through the PSO algorithm is depicted in Fig. 10. The outcomes of the optimization and the associated computational expenses for both methods are consolidated in Table 9. The optimal NPV achieved by the RF model and numerical simulation is 17% and 12% greater than the base model. The optimal NPV of the RF model is calculated to be 1.7 million USD. Instead of approximately six days needed for simulation runs, the fracturing parameters optimization with the RF model only requires less than two minutes, indicating that the proposed RF model can greatly enhance the efficiency of fracturing design.

**Table 9:** Initial range and final value of the fracture parameters.

Features	Initial range	Optimal values	
		RF model	Numerical simulation
Matrix permeability, mD	0.02	0.02	0.02
Porosity	0.1	0.1	0.1
Oil saturation	0.4	0.4	0.4
Reservoir thickness, m	18	18	18
Average fracture length/matrix length	[0.6,1]	0.92	0.89
Fracture spacing, m	[10, 30]	24	26.2
Fracture permeability, mD	[500,2000]	831	853
Average fracture height/matrix height	[0.6,1]	0.88	0.91
Optimal NPV, million USD		1.74	1.67
CUP time,s		240	618,400

## Discussions and future work

In this study, the optimization problem of fracturing parameters in petroleum engineering is solved by an equation optimization problem in mathematics, which seeks the optimal solution of the objective function under the set constraints. The RF algorithms used in the study are a model that can solve the optimization problem to maximize NPV, and the regression-like problem is transformed into an optimal solution of the objective function through the regression method of this model. The proposed workflow may potentially profit operators from accomplishing their preferred productivity and economic benefits with slight changes to fracture design or parameters influencing productivity. This study also shows some potential applications in other engineering industries, such as optimizing construction labor productivity^39^. Conventional analysis usually needs a large number of actual measured data. The proposed framework simplifies the forecast technique but offers superior computational efficiency and excellent accuracy.

A few limitations that need to be addressed include:

1. To develop and train a production prediction model, a large dataset is prepared and generated through a specific numerical simulation model, which confines the application scope of the trained model. To develop a more generic production prediction model for the target reservoir, real field data is required to be gathered and added.
2. The proposed workflow is universal for any reservoirs and the range of each feature is given as large as possible to represent broader scenarios, but the trained model is limited to the reservoirs only if their reservoir parameters fall into the feature ranges of our training dataset. Otherwise, the difference between the predicted production and real production could be remarkable.
3. PSO is computationally more efficient than other optimization algorithms, but it may fall into local optimum in high-dimension applications. Thus, an adaptive PSO technique could be considered in the workflow to promote the universality of the study in the future.

## Conclusions

We present a robust and efficient workflow to forecast production and optimize fracture parameters for unconventional oil reservoirs, by integrating reservoir simulation techniques, machine learning algorithms, and optimization methods. We estimate and compare the performance of five ML models after training the network with a dataset generated by numerical simulations. The best ML model is preferred and selected to forecast production with sufficient accuracy and efficiency. Additionally, we justified the efficacy of the trained ML model in optimizing fracturing parameters. The main conclusions are drawn as follows:

1. The established ML-based production prediction model and sensitivity analysis are employed to dissect the key influencing factors that govern the production of MFHWs within the Chang 7 tight oil reservoirs of the Panke area in the Ordos Basin.
2. The performance evaluation of the LR, SVR, GBDT, RF, and MLP models is conducted by assessing the coefficient of determination (R^2^). The yield prediction model established by the RF algorithm outperforms the other four methods in this study.
3. Compared with traditional methods such as reservoir numerical simulation, the machine learning-based method not only enables comprehensive analysis of multiple factors such as geological and fracturing that affect horizontal well production capacity but also optimizes the fracturing parameters accurately and directly in a short time to improve the fracturing production increase.

## Data Availability

The data that support the findings of this study are available from Petrochina Changqing Oilfield Company but restrictions apply to the availability of these data, which were used under license for the current study, and so are not publicly available. Data are however available from the authors upon reasonable request and with permission of Petrochina Changqing Oilfield Company. When you need this data, please contact Prof Li at weirong.li@xsyu.edu.cn.
